# The Effects of Gas Humidification with High-Flow Nasal Cannula on Cultured Human Airway Epithelial Cells

**DOI:** 10.1155/2012/380686

**Published:** 2012-09-03

**Authors:** Aaron Chidekel, Yan Zhu, Jordan Wang, John J. Mosko, Elena Rodriguez, Thomas H. Shaffer

**Affiliations:** ^1^Nemours Biomedical Research, Nemours Research Lung Center, Nemours/Alfred I. duPont Hospital for Children, 1600 Rockland Road, Wilmington, DE 19803, USA; ^2^Department of Pediatrics, Jefferson Medical College, Thomas Jefferson University, 1025 Walnut Street, Suite 700, Philadelphia, PA 19107, USA; ^3^Department of Pediatrics, Nemours/Alfred I. duPont Hospital for Children, 1600 Rockland Road, Wilmington, DE 19803, USA; ^4^Departments of Physiology and Pediatrics, Temple University School of Medicine, 3420 North Broad Street, Philadelphia, PA 19140, USA

## Abstract

Humidification of inspired gas is important for patients receiving respiratory support. High-flow nasal cannula (HFNC) effectively provides temperature and humidity-controlled gas to the airway. We hypothesized that various levels of gas humidification would have differential effects on airway epithelial monolayers. Calu-3 monolayers were placed in environmental chambers at 37°C with relative humidity (RH) < 20% (dry), 69% (noninterventional comparator), and >90% (HFNC) for 4 and 8 hours with 10 L/min of room air. At 4 and 8 hours, cell viability and transepithelial resistance measurements were performed, apical surface fluid was collected and assayed for indices of cell inflammation and function, and cells were harvested for histology (*n* = 6/condition). Transepithelial resistance and cell viability decreased over time (*P* < 0.001) between HFNC and dry groups (*P* < 0.001). Total protein secretion increased at 8 hours in the dry group (*P* < 0.001). Secretion of interleukin (IL)-6 and IL-8 in the dry group was greater than the other groups at 8 hours (*P* < 0.001). Histological analysis showed increasing injury over time for the dry group. These data demonstrate that exposure to low humidity results in reduced epithelial cell function and increased inflammation.

## 1. Introduction

Humidification of inspired gas is important for patients receiving respiratory support with nasal cannulae or mechanical ventilation. During normal breathing, the inspired gas is heated and humidified by the nasal mucosa [1]. The ACCP-NHLBI National Conference on Oxygen Therapy concluded that it was not necessary to provide routine humidification at oxygen flow rates of 1–4 L/min when environmental humidity is adequate [2].

Medical gases contain only approximately six parts per million of water vapor [3]. Administering inadequately conditioned medical gases may shift the isothermic saturation boundary (ISB) farther down the bronchial tree [4]. The ISB is the point in the airway where the inspired air in the lung reaches 37°C and 100% relative humidity (RH), and the ISB is normally located just below the carina. If the ISB shifts downward, the lower respiratory tract becomes involved in heat and moisture change [5, 6] so that the airway mucosa is at risk for mucus membrane dehydration, impaired cilia function, and retention of secretions [7], which may lead to partial or complete airway obstruction and increased incidence of infection. Previous studies demonstrated that just five minutes of respiration using ambient gas with no heating or humidification in ventilated infants resulted in a significant decrease in both pulmonary compliance and conductance [8]; cool, dry air induces a bronchoconstriction response, which is associated with muscarinic receptors in the nasal mucosa [9]. Airway cooling and drying is also a potential mechanism of exercise-induced bronchospasm. A clinical study that compared the effect of heat and moisture exchangers and heated humidifiers showed that heat and moisture exchangers generated inadequate airway humidification resulting in high incidence of endotracheal tube occlusion [10].

Heated humidification is most often used during mechanical ventilation via an artificial airway. It is believed that 30 mg H_2_O/L is the theoretical minimum humidity during invasive ventilation [11]. High-flow nasal cannula (HFNC) effectively provides temperature and humidity-controlled gas to the nasopharynx and airway. Humidification therapy plays an important role in the maintenance of the respiratory tract. However, while humidification therapy has been widely used in clinical practice, information regarding its effect on epithelial cell structure, function, and inflammatory indices is limited. 

The Calu-3 cell line (American Type Culture Collection, ATCC HTB-55; ATCC, Manassas, VA) is a well-differentiated and characterized cell line derived from human bronchial submucosal glands [12]. Calu-3 cells form high-resistance monolayers when grown on air-interfaced culture (AIC) [13–15] and have demonstrated many of the characteristics of the bronchiolar epithelium [16], which, *in vivo,* serves as the barrier layer between inspired gas and other visceral tissues. This attribute is particularly advantageous for the evaluation of airway injury and response to medical treatments and respiratory therapeutic interventions [17]. Calu-3 cells have been used for drug delivery [18], pulmonary drug disposition [19], and bacterial invasiveness studies [20]. Devor et al. [21] used the Calu-3 cell line as a model system to study the mechanisms of HCO_3_^−^ and Cl^−^ secretion, which reflects the transport properties of native submucosal gland serous cells. Other potential *in vitro* cell models have been employed for pulmonary research. Alveolar epithelial cells, such as A549 cells, lack many of the important secretory characteristics, do not form functional tight junction structure, and therefore generate very low epithelial resistance [22]. In a recent study, Stewart et al. [23] evaluated primary (human bronchial epithelial cells, HBEC) and nonprimary (Calu-3, BEAS-2B, BEAS-2B R1) bronchial epithelial cell lines as air-liquid interface-differentiated models for the *in vitro* study of asthma. These authors demonstrated that primary cells develop an inconsistent experimental phenotype and highly variable levels of transepithelial resistance (TER), thus highlighting the difficulties in utilizing primary cells for *in vitro* epithelial cell research. By contrast, Calu-3 cells formed high-resistance monolayers and expressed similar markers as compared to primary cells, suggesting that these cells may be the most suitable model cell line for air-liquid interface experiments.

In this study, Calu-3 cells served as an* in vitro* model to evaluate the effects of inspired gas humidification and high flow on the human airway epithelium. We hypothesized that levels of gas humidification would have differential effects on airway epithelial cell structure, function, and inflammatory indices from each other as well as compared with a noninterventional condition.

## 2. Materials and Methods

Calu-3 human airway epithelial cell monolayers were cultured on transwell plates with the apical surface exposed to gas and the basolateral surface exposed to culture medium. Following establishment of confluent monolayers, plates were exposed to environmental conditions as outlined below. At 4 and 8 hours, monolayer integrity and cell viability were determined by assessment of TER and CellTiter Blue Cell Viability Assay monolayer (Promega, Madison, WI). Apical surface wash fluid (ASF) samples were retrieved from additional wells for analysis of secreted inflammatory mediators. Enough wells were exposed to provide 6 observations (*n* = 6/condition) for each measured parameter. The total protein, interleukin (IL)-6, and IL-8 data were corrected by cell viability.

### 2.1. Calu-3 Cell Culture

Reagents were purchased from Invitrogen (Carlsbad, CA) and Transwell Permeable Supports from Corning Incorporated Life Sciences (Acton, MA). Calu-3 cells were cultured at 37°C and 5% CO_2_ in a 50/50% mixture of Dulbecco's Modified Eagle's Medium/Ham's F-12 (DMEM/F12) supplemented with 15% fetal calf serum, 500 u/mL penicillin, and 50 *μ*g/mL streptomycin. Cells were grown in 75 cm^2^ tissue culture flasks and split when 90–95% confluent.

Calu-3 cell air-liquid interface culture was performed as previously described [24]. Calu-3 cells were plated at 2 × 10^6^ cells/cm^2^ onto Costar Transwell inserts (0.4 *μ*m pore size, 12-mm diameter, clear polycarbonate membrane; Costar plate; Corning) treated with human type I collagen (Southern Biotech, Birmingham, AL). Apical culture medium was removed two days after plating, and monolayers were grown at an air-liquid interface and fed basolaterally with DMEM/F12 with 15% fetal calf serum. The basolateral medium was changed on alternate days. 

**Figure 1:**
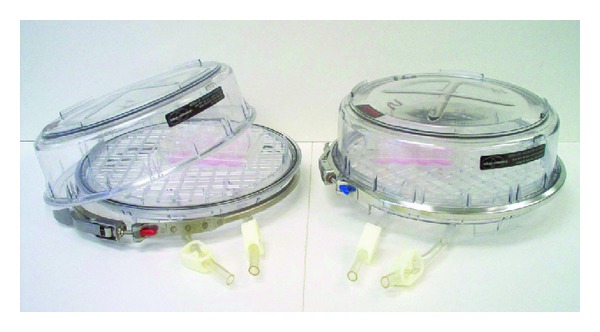
Cell-culture environmental chambers. Transwell plates were exposed to one of three levels of relative humidity: <20% (dry), 69% (noninterventional comparator), and >90% (HFNC) for 4 and 8 hours with 10 L/pm of room air at 37°C.

After 11 days of transwell culture, full confluence was verified by measurement of TER with STX2 electrodes and an epithelial volt ohm meter (World Precision Instruments, Sarasota, FL). Calu-3 monolayers that exhibited TER values of at least 800 to 1,000 ohm*·*cm^2^ were used for the experiments. For all conditions, one additional plate was placed in an incubator at RH 69% and 37°C as a noninterventional comparator group. Two plates of Calu-3 monolayers were placed in modular incubator chambers (MIC-101; Billups-Rothenberg, Del Mar, CA) and exposed to one of two levels of RH: less than 20% and more than 90% for 4 or 8 hours with 10 L/min of continuous room air flow at 37°C. To achieve the RH of less than 20%, the gas from 21% FiO_2_ tank was flushed through the chamber using a Servo O_2_-air 960 mixer (Siemens-Elema, Solna, Sweden) for 4 or 8 hours at a flow rate of 10 L/min in an incubator at 37°C. The HFNC (Vapotherm, Stevensville, MD) provided the gas at a flow rate of 10 L/min and maintained the temperature at 37°C and the RH at 90%. Chamber oxygen level was monitored with an oxygen analyzer (MAXO_2_; OM-25AE; Maxtec, Salt Lake City, UT). The modular incubator chamber was sealed, but the O ring was not compressed by a stainless steel ring clamp. This would allow the chambers to open in case the pressure inside exceeded atmospheric pressure. Pressure was confirmed by a Rüsch pressure monometer (Teleflex Medical, Duluth, GA), and the humidity was monitored by a humidity thermometer (Fisher Scientific, Cat no. S66279) inside the modular incubator chamber (Figure 1).

At each experimental time-point, monolayers were used for measurement of TER (*n* = 6/condition) or ASF collection (*n* = 6/condition). Six monolayers were then used for assessment of cell viability and cytomorphometry (*n* = 3/condition).

The ASF samples were collected as previously described [15]. Monolayers were washed twice with 140 *μ*L of normal saline. Washes from each insert were combined for a total volume of 280 *μ*L and centrifuged for 15 min at 13,000 rcf and 4°C to remove debris. Supernatants were stored in aliquots at −70°C for subsequent assays. 

### 2.2. Measurement of Cell Viability

CellTiter Blue Cell Viability Assay (Promega, Madison, WI) was performed to estimate the number of viable cells and determine cell viability by the intensity of fluorescence. This assay is based on the reduction of resazurin to resorufin by living cells. Triplicate wells were set up without cells to serve as the negative control to determine background fluorescence. The monolayers exposed to RH 69% and 37°C served as a noninterventional comparator group. 200 *μ*L of medium and 40 *μ*L of CellTiter Blue reagent were added to the apical side of the transwell insert and 720 *μ*L of medium to the basolateral side. The fluorescence was recorded using a plate-reading fluorometer at 550/590 nm.

### 2.3. Measurement of TER

The TER measurements were made using STX2 electrodes and an epithelial volt ohm meter (World Precision Instruments). At baseline and after treatment, 0.5 mL of medium was added apically and 1 mL of medium was added basolaterally, and electrodes were inserted into each pool of medium to measure TER. 

### 2.4. Measurement of Total Protein

Total protein concentration was measured in duplicate using DC protein assay kit (Bio-Rad, Hercules, CA). Bovine serum albumin (standard II) was selected as a reference standard. Absorbance was measured at 655 nm.

### 2.5. Measurement of IL-6 and IL-8 Levels in Calu-3 ASF

The ASF levels of IL-6 and IL-8 were measured in duplicate by quantitative enzyme-linked immunosorbent assay (ELISA) using human IL-6 and IL-8 Quantikine ELISA kits (R & D Systems, Minneapolis, MN). The test sensitivity for respective immunoassays was 0.039 pg/mL for IL-6 and 10 pg/mL for IL-8. Interassay and intraassay coefficients of variance were less than 10%. IL-6 and IL-8 data were corrected for cell viability. 

### 2.6. Histology and Cytomorphology of Calu-3 Cells

Cytospin slides were prepared using 100 *μ*L of single-cell suspensions and stained for cell differentiation using Kwik-Diff solutions (Thermo Electron, Pittsburgh, PA). Cytomorphology was evaluated in a semiquantitative manner as the ratio of abnormal cells to total cells. Abnormal cells were defined by the following characteristics: (1) abnormal cellular appearance, (2) swollen nuclei, (3) intracellular and nuclear vacuoles, and/or (4) diffuse cytoplasm and cellular debris [25]. Two-hundred cells were evaluated for each experimental condition in the following fashion. Microscope slides were evaluated by the scorer in a blinded fashion to the experimental condition by randomly selecting four fields for visual observation. Fifty individual cells from each of four high-power fields were scored and only after each slide was scored was the experimental condition unblinded.

### 2.7. Statistical Analysis

Statistical analysis was performed using Statistical Package for the Social Sciences (SPSS, Version 17, IBM, Armonk, NY) software. All data were expressed as mean ± SEM. Differences between conditions for each parameter were analyzed using two-way analyses of variance (ANOVA). Where applicable, one-way ANOVA was run across different treatments at each time interval (i.e., 24 and 72 hours), and post-hoc analyses were done using Bonferroni comparisons. Significance was accepted at *P* < 0.05. At least six samples were used for each group.

## 3. Results

**Figure 2:**
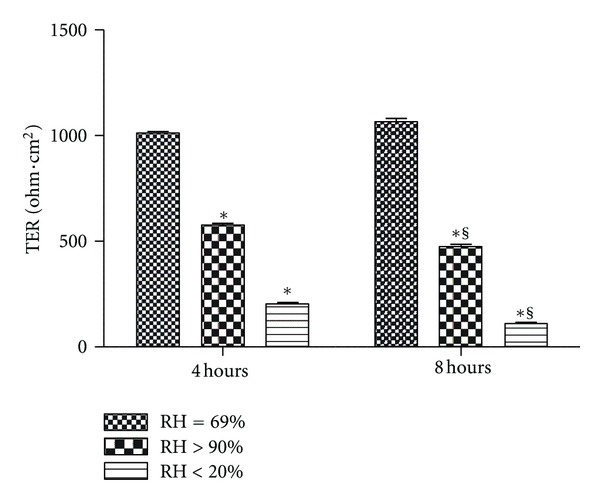
Transepithelial resistance (TER) for Calu-3 monolayers exposed to one of three levels of relative humidity: <20% (dry), 69% (noninterventional comparator), and >90% (HFNC). TER was less than the noninterventional comparator group in the HFNC and dry groups (*P* < 0.001). Data are mean ± SEM. *Group effect (*P* < 0.001). ^§^Time effect (*P* < 0.001).

After 11 days culture, Calu-3 cells developed a baseline TER of 1105.86 ± 43.8 ohm*·*cm^2^ for all monolayers before the experiments. Calu-3 monolayers exposed to the gases with RH of less than 20% (dry group) and more than 90% (HFNC group) showed decreased TER (*P* < 0.001) over time. The TER in the noninterventional comparator group remained constant at the same level at 4 and 8 hours (Figure 2). Two-way ANOVA demonstrated an interaction for humidification and time of exposure (*P* < 0.001) whereby the TER did not change over time in the noninterventional comparator group but decreased in both the HFNC and dry groups. Post-hoc analysis revealed that the HFNC and dry groups were less than the noninterventional comparator group independent of time (*P* < 0.01). In addition, the TER of monolayers exposed to high humidity decreased less than those exposed to low humidity at both time points (*P* < 0.001).

**Figure 3:**
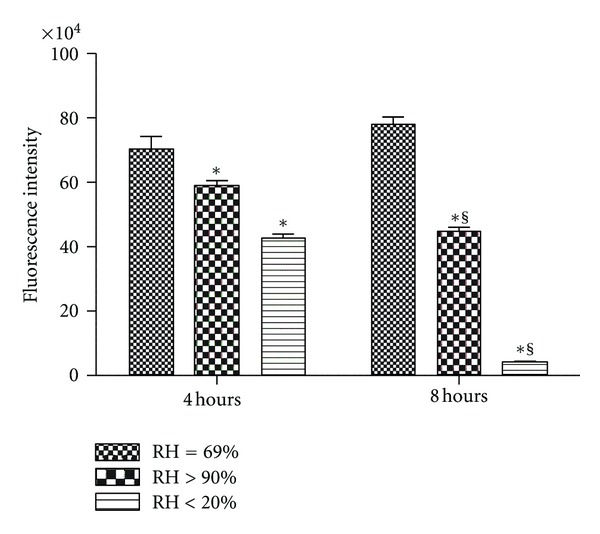
Cell viability for Calu-3 monolayers exposed to one of three levels of relative humidity: <20% (dry), 69% (noninterventional comparator), and >90% (HFNC). CellTiter Blue cell viability assay was performed to estimate the number of viable cells and determine cell viability by the intensity of fluorescence. Cell viability decreased over time (*P* < 0.001) for HFNC and dry groups. Data are mean ± SEM. *Group effect (*P* < 0.05). ^§^Time effect (*P* < 0.05). *n* = 6/condition.

Cell viability also decreased over time (*P* < 0.001) for both of the intervention groups with a similar pattern noted as for the TER. Cell viability remained unchanged in the noninterventional comparator group, decreased 42.54% in the HFNC group, and decreased substantially (94.60%) in the dry group (Figure 3). 

**Figure 4:**
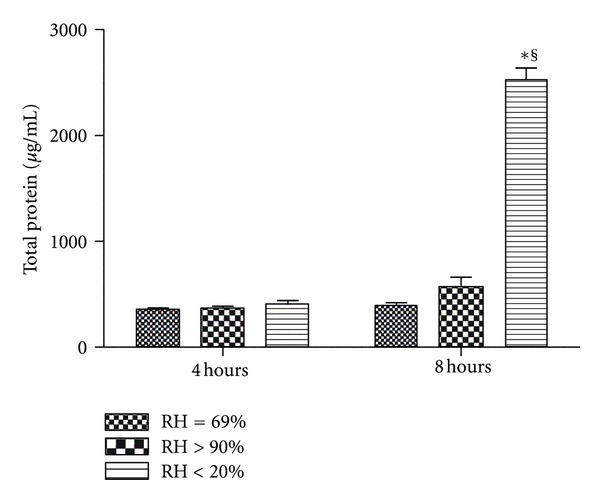
Total protein concentration in apical surface wash fluid from Calu-3 monolayers exposed to one of three levels of relative humidity: <20% (dry), 69% (noninterventional comparator), and >90% (HFNC). Secretion of total protein increased at 8 hours in the dry group (*P* < 0.001), but there was no difference between the noninterventional comparator group and the HFNC groups (*P* > 0.05) and no difference between groups at 4 hours (*P* > 0.05). Data are mean ± SEM. *Group effect (*P* < 0.05). ^§^Time effect (*P* < 0.05). *n* = 6/condition.

Secretion of total protein in the dry group ASF samples increased at 8 hours (*P* < 0.001), but there was no significant difference between the noninterventional comparator group and the HFNC groups (*P* > 0.05) and no significant difference between any of the groups at 4 hours (*P* > 0.05) (Figure 4).

**Figure 5:**
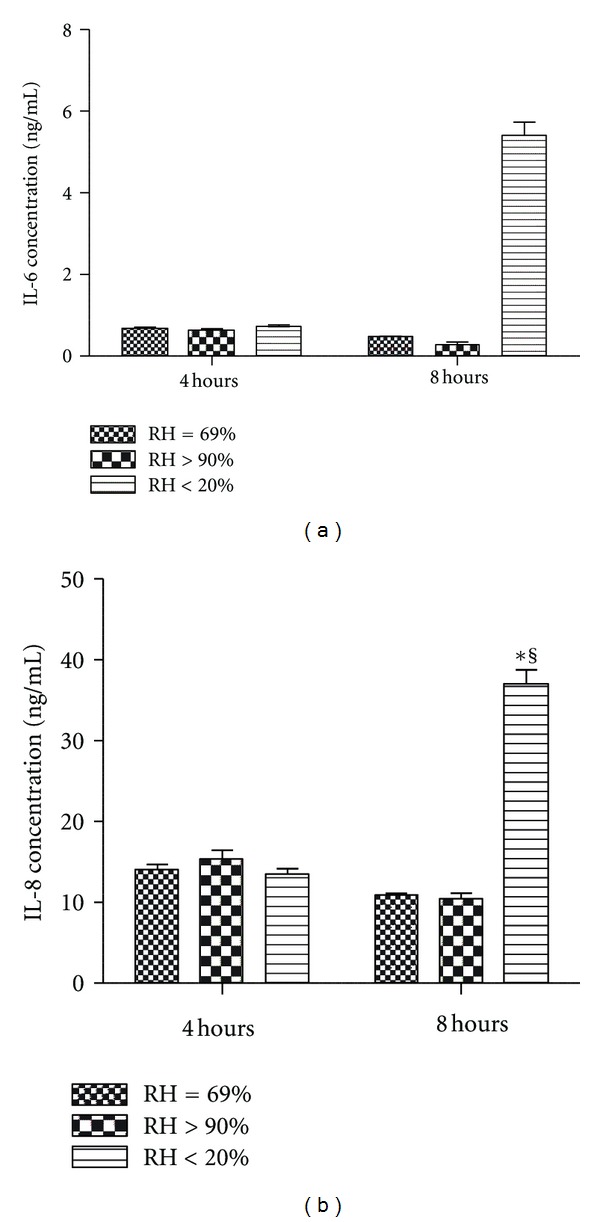
Proinflammatory mediator concentration in apical surface wash fluid from Calu-3 monolayers exposed to one of three levels of relative humidity: <20% (dry), 69% (noninterventional comparator), and >90% (HFNC). Secretion of interleukin (IL)-6 (5A) and IL-8 (5B) in the dry group was greater than the noninterventional comparator group and the HFNC groups at 8 hours (*P* < 0.001), with no difference between the noninterventional comparator group and the HFNC groups (*P* > 0.05) and no difference between any groups at 4 hours (*P* > 0.05). Data are mean ± SEM. *Group effect (*P* < 0.05). ^§^Time effect (*P* < 0.05).

Secretion of the inflammatory mediators IL-6 (Figure 5(a)) and IL-8 (Figure 5(b)) in the dry group was greater than in both the noninterventional comparator group and the HFNC groups at 8 hours (*P* < 0.001), with no significant difference noted between control and HFNC groups (*P* > 0.05) and no significant difference between any of the groups at 4 hours (*P* > 0.05). 

**Figure 6:**
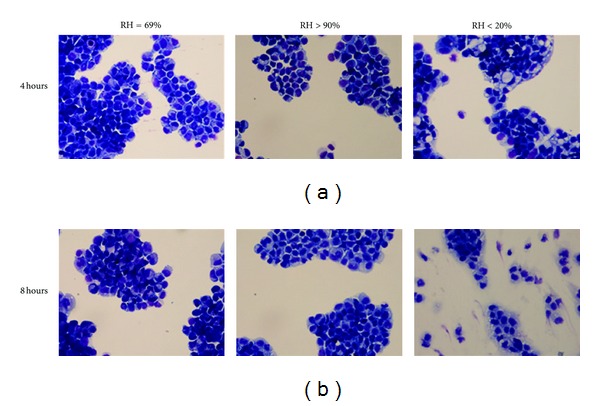
Cytomorphological examination of Calu-3 cell monolayers exposed to one of three levels of relative humidity: <20% (dry), 69% (noninterventional comparator), and >90% (HFNC). Representative cytomorphological examination of Calu-3 cells for both the noninterventional comparator group and the HFNC group demonstrated normal morphology. At 4 and 8 hours, the dry group showed abnormal cellular appearances, swollen nuclei, intracellular and nuclear vacuoles, diffused cytoplasm, and cellular debris. All cytospins were examined by light microscopy at 40x magnification.

**Figure 7:**
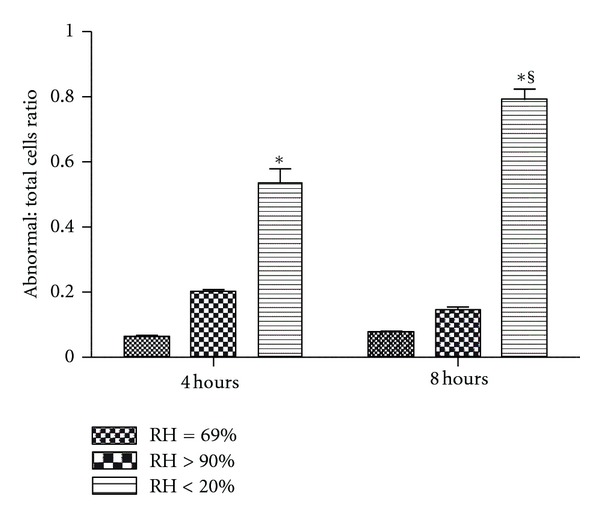
Semi-quantitative assessment of cell morphology as the ratio of abnormal cells to total cells. Histological analysis did not show injury for the noninterventional comparator or the high flow nasal cannula groups at 4 and 8 hours, whereas the dry group demonstrated increasing injury over time. Data are mean ± SEM. *Group effect (*P* < 0.05). ^§^Time effect (*P* < 0.05). *n* = 6/condition.

At 4 hours and 8 hours, the monolayers of the dry group demonstrated a dry, rough, and granular appearance upon visual (naked eye) inspection. Representative histological photomicrographs at 4 and 8 hours are presented in Figure 6. The dry group showed abnormal cellular appearances, swollen nuclei, intracellular and nuclear vacuoles, diffused cytoplasm, and cellular debris. Semi-quantitative assessment of cell morphology (Figure 7) did not show injury for the noninterventional comparator group or HFNC groups at 4 and 8 hours, whereas the dry group demonstrated increasing histological evidence of injury over time.

## 4. Discussion

In this study, we used Calu-3 cells in an *in vitro* model to evaluate the effects of inspired gas humidification on the structure and function of cultured human airway epithelial cells. This study shows the effectiveness of maintaining a specific homeostatic temperature and level of humidity to optimize cell function in Calu-3 airway monolayers. These experiments demonstrate that humidification therapy is beneficial to the maintenance of cellular structure and function in that it enhances Calu-3 monolayer integrity, betters cell viability and morphology, and reduces airway epithelial inflammation compared to cells exposed to a dry condition over an 8-hour exposure time. 

In this study, TER was measured for examination of the integrity of the monolayers [26] and the formation of functional intercellular tight junctions [27]. During the experiment, low humidity (less than 20%) decreased TER and cell viability over time compared with both HFNC-treated and noninterventional comparator groups. There was a partial deterioration in TER even in the HFNC-treated monolayers. This may be due to shear forces to which these monolayers are exposed, independently of gas humidification. A study showed that gas delivered by HFNC was more humid than nasal cannula and continuous positive airway pressure; however, the higher pressure and resistance delivered by the HFNC system may cause excessive expiratory pressure loading [28] and may also result in airway injury by this mechanism. We attempted to correct for pressure loading of the monolayers in our experiments by leaving the modular incubator chamber closed but unsealed by the pressure gasket.

These findings were also consistent with the findings in the histological analyses. Calu-3 cells in the dry group demonstrated disrupted morphology at 4 and 8 hours. Both the noninterventional comparator group and HFNC groups maintained normal cell morphology over 8 hours. Histological analysis did not show injury for the noninterventional comparator group or HFNC groups at 4 and 8 hours, whereas the dry group demonstrated increasing injury over time. In the current experiments, the decreased TER and cell viability indicated a loss of intracellular adhesion integrity, cellular membrane damage, and impaired cell metabolism after being exposed to low humidity for 8 hours. 

In the dry group, the oxygen tank delivered the gas directly without adequate humidification at a flow rate of 10 L/min. This exacerbated the water evaporation from the surface of the monolayers in the modular incubator chamber. We speculate that the low humidity resulted in dehydration of Calu-3 cells, which was evident by the dry, rough, and granular appearance of the monolayers at 4 and 8 hours. The desiccation of the monolayers may have contributed to the disruption of intracellular adhesions and acute (necrotic) cell death with low humidity exposure.

Previous studies have shown that ASF and protein secretions in Calu-3 cultures are similar to those from primary airway cultures [29–31]. Total protein is a marker of cell membrane dysfunction and injury and is observed in noncardiogenic pulmonary edema states of acute lung injury in the clinical arena. In the current study, the secretion of total protein for Calu-3 monolayers increased over time in the dry group at 8 hours. There was no group effect between groups at 4 hours. These results demonstrate that low humidity induced higher protein secretion in the Calu-3 cells. We did not evaluate the mechanisms for this finding but speculate that it is also related to a nonspecific response to cell injury. 

The pro-inflammatory mediators IL-6 and IL-8 are chemoattractant factors, which, *in vivo,* result in neutrophil infiltration and an exaggerated systemic inflammatory response [32]. IL-6 is a pleiotropic cytokine that is produced at sites of affected tissue. IL-8 plays an important role in the initiation and propagation of the inflammatory cascade and lung injury, primarily by neutrophil recruitment [30, 31, 33, 34]. The exposure of Calu-3 cell monolayers to low humidity (less than 20%) triggered Calu-3 cells to secrete increased levels of IL-6 and IL-8 compared with the noninterventional comparator group and HFNC group by 8 hours. In previous studies, we found that Calu-3 cells respond to pro-inflammatory stimuli, such as hyperoxia and pressure, in a graded fashion [25]. The results of the current experiments also suggest that airway drying causes an inflammatory response in a similar fashion. In addition, the inflammatory response observed in Calu-3 cell preparation was consistent with our previous animal study findings, which showed that physical and biological stimuli in the airway causes both systemic and local inflammation *in vivo*. In this regard, repeated intubation without mechanical ventilation and hyperoxia resulted in elevated levels of IL-6 in the tracheal tissue, aspirates, and plasma in a neonatal porcine model [35]. In another study, it was shown that after 4 hours of positive mechanical ventilation, secretion of heat shock protein (HSP70), a marker of tissue injury and inflammation modulation, increased in the tracheal wash fluid from a neonatal lamb airway [36]. 

In conclusion, after 4 hours, there was a significant detrimental effect of low humidification as a function of time on Calu-3 monolayers, TER, and cell viability for the dry group. After 8 hours, low humidity triggered Calu-3 cells to secrete increased levels of total protein, IL-6, and IL-8. These effects were ameliorated or eliminated with the use of HFNC. The study demonstrates findings that may have potential clinical relevance of inspired gas humidification for even short periods of time since exposure to low humidity results in worsened epithelial cell function and in inflammatory indices.
